# The Obstetric Memoirs and Contributions of James Y. Simpson, M. D., F. R. S. E.

**Published:** 1855-12

**Authors:** 


ART. V.—*The Obstetric Memoirs and Contributions of* JAMES Y. SIMPSON, M. D., F. R. S. E., Professor of Midwifery in the University of Edinburg, etc., etc. Edited by W. O. PRIESTLEY, M. D., Edinburg, formerly Vice-President of the Parisian Medical Society, and HORATIO R. STORER, M. D., one of the Physicians to the Boston Lying-in Hospital, Member of the Medico-Chirurgical and Obstetric Societies of Edinburg, etc., etc., etc. Vol. I. Philad.: J. B. Lippincott & Co. 1855. 8vo. pp. 756.

The author of these Memoirs is one of the most distinguished of living obstetricians, and one of the most prolific medical writers of the day. The volume before us, comprising nearly eight hundred large pages—a beautifully executed volume let us remark at the beginning—constitutes but little more than half of what he has written, and yet Dr. Simpson is under forty-five years of age. Few men have innovated so largely or so successfully upon obstetric practice as he has done during his comparatively brief career. We enumerate a few of these innovations.

In 1844 Dr. Simpson, in certain cases of placenta previa, proposed the extraction of the placenta before the delivery of the child.

In 1847 he proposed the operation of turning as an alternative for craniotomy, and in the same year recommended the use of anæsthetics in labor.

These proposals have met with the assent of a large proportion of the profession, and their adoption, we are persuaded, has not only averted an incalculable amount of suffering but saved the lives of many a mother.

Dr. Simpson, in 1843, proposed the sound in uterine diagnosis; and the same year he suggested the treatment of displacements of the womb by intra-uterine pessaries. The year following, in cases of obstructive dysmenorrhœa, he recommended the incision of the cervix uteri; and in 1849 he proposed the employment of sponge-tents to expose intra-uterine polypi.

These propositions have not been so generally concurred in by obstetricians, but it is due to Dr. Simpson to say that many sound practitioners, perhaps a majority, have sanctioned them. It is unquestionably true that the sound is necessary to the diagnosis of certain female diseases, and the tent to the cure of others. The violence with which some of his propositions have been assailed has appeared to us unaccountable, except upon grounds of personal hostility. It seems that such personal enmity has operated in at least one quarter, and there is strong reason to believe that opposition to the author has imparted unreasonable warmth to the opposition to his opinions. Weak as such a course is, it is one into which men do occasionally suffer themselves to be betrayed. Improvements and discoveries have often been rejected on no better ground than that of hostility to their authors. There are those who will decline the use of a good remedy because an obnoxious rival employs it. No doubt some of our readers will be able to call to mind instances similar to the one recounted by Dr. Simpson's American editor in the preface to his work, which is as follows:—

" Dr. Robert Lee, of London, though himself a Scotchman, has for many years been Dr. Simpson's inveterate opponent, and has successively attacked every proposition of any importance he has made since 1840—the date of their competing for the chair Dr. Simpson now holds in the University of Edinburg. To several of these attacks Dr. Simpson has replied at length; the remainder he has probably considered beneath his notice. Among others, Dr. Simpson's proposal of the intra-uterine passary could not, of course, remain unnoticed, and on various occasions and in various connections Dr. Lee has subjected it to unmeasured ridicule, and at no time has he been at all temperate in his use of language. He has attacked Dr. Simpson at all times and seasons. Within even a year he has published in one of the London journals a most furious tirade upon anesthetics in labor. He has written alike in his own name and anonymously; and I think no one can systematically follow out the whole details of his warfare, as I have done, and still consider Dr. Lee an earnest physician laboring only for truth's sake. I regret to say this, and have done so with great reluctance. I know that it will give Dr. Simpson pain. But I must add that Dr. Lee's chief works on obstetrics, which formerly gained for him such reputation as a practitioner and as a writer, are now becoming considered entirely unreliable. His famous system of uterine nerves has been proved by Dr. Snow Beck, from the same identical specimens, not to exist; and the honors conferred upon him by one of the highest Societies in Great Britain,* have been formally and ignominiously cancelled."

The subjects treated of in this volume are arranged under three heads, the first of which comprises forty-three papers on the Special Pathology of the unimpregnated Female, the second embraces five on the Physiology and Pathology of Pregnancy, and the third thirty-three on Natural and Morbid Parturition. Many of these are short, extempore papers, but others are long and highly elaborate, and will maintain their places among the best productions of the day on obstetric medicine. As in the space afforded by a single number of our Journal it would be impossible to review all the subjects treated of by Prof. Simpson, we shall confine ourselves to an examination of some of the more important papers which especially characterize the views and practice of the author.

In the first, the subject of the general diagnosis of uterine disease is considered at great length, and in it the reader will find expressed many of the views respecting the pathology of the uterus upon which much of the author's peculiarity of practice is founded. He treats of the subject under two heads: the *symptoms* and the *signs* of the uterine disease. In the former, he refers to the five following sources or varieties of information:—

"1. *Derangements in the Functions and Vital Condition of the Uterus itself*, as indicated by the quantity, character, periodicity, etc., of the menstrual and mucous secretions of the organ; by the occurrence of morbid uterine or vaginal discharges—as blood,serous fluid, pus, etc.; by the existence of morbid sensations in the region of the uterus—such as different modifications of pain, intermittent or continuous, feelings of heat, weight, tension, bearing down, etc.; and if the patient be married, by the reproductive powers being affected, as shown by sterility, the recurrence of abortions, premature labor, and the like.

"2. *Dynamic Symptoms in neighboring Pelvic Organs*, more especially affections of the rectum and bladder, and of branches of the vessels and nerves passing through the pelvis. In this division are included pains about the bladder or rectum, in the groins,

* The Royal Society of London.

along the crest of the ilium, and along the course of the crural and sciatic nerves; intermittent pains in the lower part of the abdomen: derangement of the functions of the rectum or bladder, producing either constipation, or difficult or painful defæcation on the one hand, or too frequent micturition, dysuria, retention or incontinence of urine on the other.

"3. *Sympathetic Pains in different and distant Parts of the Body*, including pain in the mammæ, along the lower extremities; in the loins, and at points along the course of the spinal column; in the parietes of the thorax or abdomen; on one or other side, and especially under the left breast; along the course of the colon; under the margin of the ribs; increased by any causes which tend to an increased action of the uterus, such as the erect position, menstruation, etc.

"4. *Derangements of Functions in Distinct Organs*, including affections of the kidneys, intestinal canal, liver, lungs, nervous system, and skin.

"5. *States of General Constitutional Derangement.* These comprise more particularly febrile, cachectic, and anæmic conditions."

The above are the chief symptoms, local, constitutional, and sympathetic, of diseases of the uterus, but whether always originating in uterine affections, or in diseases of other parts involving the uterus secondarily, is a question which we shall not now stop to discuss.

Dr. Simpson gives the following summary of the *signs* of uterine disease:

"1. The external or abdominal examination of the patient by sight, touch, auscultation, and percussion.

"2. The tactile examination of the uterus, ovaries, etc., by the vagina or by the rectum.

"3. That most important mode of diagnosis, viz: the simultaneous combination of the external and internal modes of tactile examination.

"4. The use of the speculum.

"5. The use of the uterine sound.

"6 The use of sponge-tents, with the view of dilating the os uteri, so that the finger can be introduced into the cavity of the cervix or cavity of the body of the organ.

"7. The microscopic and chemical examination of the discharges from the uterus and vagina.

"8. The employment of the exploring needle in cases of fluid collections, in order to ascertain the contents of such collections; and

"9. The adoption of anæsthetic agents to relax the abdominal parietes, and enable us to practise the different modes of examination, in cases of excessive or neuralgic tenderness of the abdominal surface or vagina, etc."

In this paper Dr. Simpson insists with great earnestness upon the paramount certainty of physical diagnosis, and the utter insufficiency of rational symptoms, in the discrimination of uterine disease. This preference for physical means of diagnosis has perhaps led the author to the undue preference for physical methods of cure with which he has been charged by many of his brethren. He depicts forcibly in the following passage the evils of "exclusiveness," and yet seems not to be aware that in relying so exclusively upon physical means of diagnosis he is running into the very error he condemns:—

"Since the diseases of the uterus and its appendages have of late years attracted so much more the attention of the profession than they formerly did, one grevious error has been committed in their study and investigation. The error I allude to is the error of exclusiveness. Formerly many practitioners seemed to look upon all diseases of the uterus as diseases indicative of debility; and they treated almost every one of them with muriate of iron and other chalybeate preparations, sometimes adding, where there was any discharge, the local employment of astringent or tonic injections. I fear that even yet you will find some old practitioners treating their uterine cases upon this sole principle. Then a second set of pathologists would, if we may judge from their writings, seem to suppose that all diseases of the uterus are marked by, if they do not consist of, congestion and engorgement of blood; and that they are to be remedied by the remedies applicable to their state. A third set, again, look upon the general run of uterine cases as almost invariably inflammatory in their nature, and imagine that we are sure, or almost sure, to find in every case, inflammation, or some of its results, as ulceration, purulent discharges, etc. A fourth set would seem to fancy all uterine ailments to be produced by some mechanical displacement or dislocation of the uterus, and to consist of prolapsus, versions, and flexions of this organ upon itself and upon the neighboring parts. Again, there are some practitioners—one in particular, in immense practice in America—who would appear to believe that the affections of the uterus are fundamentally nervous or neuralgic disorders, and that they are always to be treated by the local inunction upon the cervix uteri of morphia, aconite and similar sedatives. Another section of pathologists imagine the so-called uterine diseases are, after all, not uterine, but ovarian; and that ovarian irritation and inflammation is actually the source and origin of much of the suffering that is imagined to be uterine in its seat. Lastly, for it is needless to extend this tedious enumeration, you will find another set enjoying the belief, that these supposed uterine or ovarian diseases are not at all uterine or ovarian in their origin, but in reality diseases of the general system; they may not deny that local affections do occur in the uterus and ovaries, but these local affections are, in their opinion, results and effects of some more general or constitutional disease of the nervous system or of the economy at large."

The memoir on the uterine sound is an elaborate and important one, and concludes with the following summary ot the chief purposes which the instrument is calculated to fulfil:

"1. The sound increases to a great degree our power of making a perfect and precise tactile examination of the fundus, body, and cervix of the uterus, by enabling us to bring these portions of the organ successively into the most convenient position for external or internal examination or manipulation.

"2. The previous introduction of the instrument facilitates and simplifies the subsequent visual examination of the cervix uteri with the speculum by lessening the difficulty which sometimes arises of catching the os and cervix uteri in the upper or internal extremity of the instrument.

"3. By its use we may, in many instances of pelvic and hypogastric or abdominal tumors, ascertain the connexion or non-connexion of these tumors with the uterus. Thus, when the tumor is uterine, the instrument, when passed into the uterine cavity, enters, as it were, more or less into the very structure of the morbid mass, and the tumor and instrument afterwards reciprocally move in exact correspondence with each other. When, however, the tumor is not uterine—(1,) the uterus may be retained in its situation with the sound, and then, by means of the hand above the pubis, or by some fingers in the vagina, the tumor, if unattached to the uterine tissues, may be moved away from the fixed organ; or (2,) the tumor being left in its situation, it may be possible to move away the uterus from it to such an extent as to show them to be unconnected; or (3.) instead of keeping the uterus fixed and moving the tumor, or fixing the tumor and moving the uterus, both may be moved simultaneously, the uterus by the bougie, and the tumor by the hand or fingers, to opposite sides of the pelvis, to such an extent as to give still more conclusive evidence of the fact.

"4. The uterine bougie is capable of affording valuable diagnostic information, by enabling us to measure the length of the uterine cavity, as in the following cases, in which it is abnormally increased—(1,) Morbid permanence of the state of puerperal hypertrophy; (2,) Normal elongation of the puerperal uterus as a sign of delivery; (3.) Increased length in metritic and congestive hypertrophy of the body of the uterus; (4.) Longitudinal hypertrophy of the uterus, and especially of the cervix; (5.) Enlargement of the uterus and uterine cavity from the growth of fibrous tumors in the parieties of the organ; (6.) Enlargement and distension of the uterus from polypi; (7.) Elongation of the uterus in hernia of the organ. The uterine bougie affords information in the following cases, in which the uterine cavity is abnormally diminished; (1.) Preternatural shortness of the uterus from original malformation; (2.) Shortening of the uterine canal from stricture or partial obliteration; (3.) Diminution of the depth of the uterine cavity from inversion of the organ."

The papers on polypi of the uterus are highly interesting. Some of the points started are the following: The employment of the uterine sound in cases of polypi growing from the lips of the cervix, which Dr. Simpson believes will afford information of the greatest value; the employment of sponge tents for the mechanical dilatation of the cervix in cases of intra uterine polypi to such an extent as to admit of the introduction of a finger into the uterine cavity, for the purpose of exploration and diagnosis; and the advantages of the excision of large pedunculated polypi over deligation. This operation the author regards as being not only less hazardous and more easily performed, but safer than the one of excision by ligature. We concur in this opinion, and have no doubt that it will be generally adopted by the profession.

An important series of papers on malignant diseases of the uterus follows, in one of which the nature of the cauliflower excrescence is considered:

"The pathological nature of this variety of morbid growth, has given rise to a considerable difference of opinion among physicians. Drs. Gooch, Hooper, Davis, and Lee regard it as truly cancerous in its character; others—as Drs. Clarke, Burns, and Waller—consider it as a morbid tissue, not necessarily of a malignant or carcinomatous nature. A number of circumstances appear to me to show that, in reference to at least the first stage of cauliflower excrescence, the opinion of these latter authors is probably correct. The occurrence of the disease in some cases as early as the twentieth year of life; its occasional shrinking and almost total disappearance upon the application of a ligature, or after death; the frequent slowness of its general progress during life; the apparent absence of diseased deposits in the neighboring tissues and parts upon the dead body; and, above all, the alleged restriction, and even complete removal, of the tumor, in one or two instances, by the use of astringent applications and other simple means, form so many circumstances strongly pointing to the opinion that, in the earlier part of its progress, the tumor cannot be regarded as of a carcinomatous character."

The above favorable view of the nature of the disease is hardly sustained by the following remarks on the same subject:

"But whatever view we may take of the primary nature of the cauliflower excrescence of the cervix uteri, we have sufficient evidence for believing either that this disease has been often confounded with carcinomatous or medullary fungus from the cervix uteri, from the want of adequate diagnostic marks to distinguish them; or that, though non-malignant in its commencement, the cauliflower excrescence may, like some other local benign growths, become the seat of carcinomatous deposit and malignant action during its progress. Thus it has been found by Gooch and Madame Boivin to return again in a malignant form, after its imperfect removal by the ligature or knife. In an instance mentioned by Dr. Davis, its removal was followed, after the lapse of a considerable period, by its reproduction, and ultimately by carcinomatous ulceration; and in two cases that occurred to Professors D'Outrepont and Siebold, in which large tumors having a cauliflower form were found affixed to the cervix uteri during parturition, the neighboring uterine tissues, as well as the contiguous structures of the bladder and uterus, were found in a carcinomatous state upon the post-mortem dissection. In another case, in which Michaelis excised what he terms a *fungus medullaris* with a cauliflower appearance, from the anterior lip of the uterus during labor, the posterior lip of the organ afterwards degenerated, and cancer of the stomach ultimately supervened."

We apprehend that this excrescence of the uterus is always malignant in its character, all its varieties being referable to the epithelial or the medullary cancer. Certainly it bears the closest resemblance to the former, which in one stage is a malignant, and in another appears to be a non-malignant, disease. Mr. Paget, in his admirable lectures on Surgical Pathology, has the following remarks on the papillary tumors of the uterus, which Virchow had described as of three kinds:

"It is evident from his description that the cauliflower excrescence, in the two conditions distinguished by him, illustrates the usual history of the most exuberant epithelial cancers; it might be taken as the principal example of the group. That which he calls the '*simple papillary tumor*,' is an excessive papillary outgrowth of epithelial cancer; the latter stage of the same, when it passes into '*cancroid*,' is the usual extension of such a cancer into deeper parts—a continuous growth of the same thing in a new direction; for the papillary structures—composed, as Virchow says, of epithelial cells with blood vessels and a very little connective tissue—are the essential characters of the epithelial cancerous outgrowths; and I believe that the same composition has never been seen in any papillary or warty growths that did not, if time were allowed, proceed to the formation of epithelial structure in the deeper parts, and thence through the usual progress of malignant disease."

Cauliflower conforms to this description, in its earliest stage being of a simple, benign nature, but in its further progress assuming a character of malignancy.

Dr. Simpson mentions the following forms of disease as furnishing cases for the excision of the cervix uteri:

"1. Great morbid hypertrophy, by elongation of the vaginal portion of the cervix uteri.

"2. Corroding ulcer, when limited to the lips of the cervix, and pathologically identical with the form of lupus of malignant ulcer so well known on the face; and

"3. Circumscribed and local forms of carcinomatous disease, or excrescence of the lips and lower segment of the cervix uteri.

We confess we should not perform the operation in any of these cases. Morbid hypertrophy we should not esteem an affection grave enough to justify so serious a procedure; and in cancer we should deem it ineffectual.

Dr. Simpson attaches great pathological importance to retroversion of the unimpregnated uterus. The following is a summary of his leading doctrines and practical deductions:

"1. That the disease had generally been considered to be rare, merely in consequence of the want of a proper and easy means of detecting it; but that since the author had made this discovery, he had found it to be one of the most common and frequent displacements and affections of the unimpregnated uterus;

"2. That the functional symptoms of this lesion are of a very varied and uncertain character, sometimes being either few or altogether absent, and sometimes extremely severe and distressing. That when present, they are more or less perfect imitations of the secondary phenomena of pregnancy, such as dyspeptic and hysterical symptoms, neuralgic pains in the mammæ, in portions of the vertebral column, or in the parietes of the chest or abdomen. Mechanical irritations and symptoms in neighboring organs, more especially of the rectum and bladder, giving rise to constipation or impeded defæcation on the one hand, or to dysuria, retention, or incontinence on the other. Weight, tension, and bearing down in the regions of the uterus and rectum, with dragging at the loins and in the region of the uterine ligaments. Pains stretching down one or both lower extremities. Inability to bear carriage exercise, whilst walking and standing speedily produce fatigue. In some cases menstruation is not morbidly altered, but in others it is either suppressed, painful, or excessive. Leucorrhœa is sometimes absent, and at other times present; and when pregnancy occurs, abortion is apt to take place; but in some cases the uterus is spontaneously rectified, and utero gestation proceeds to the full time. Usually it interferes with the function of conception, and is often a cause of sterility;

"3. That with regard to its physical signs, the employment of sight by means of the speculum in no respect assists our diagnosis; but that, on tactile examination, a solid tumor is to be felt behind the cervix uteri, between the uterus and rectum, and the same firm mass may be felt through the anterior wall of the bowel, in making an anal examination; and that the most simple and certain method of diagnosis consists in the introduction of the uterine sound, which, by following the curvature or reflexion of the uterus, can be made to enter and traverse the centre of the supposed tumor;

"4. That the most frequent abnormal conditions with which it is apt to be confounded are—1. Pregnancy. 2. Fibrous and other tumors in the posterior wall of the uterus. 3. Ovarian tumors in their earlier stages. 4. Pelvic cellulitis. 5. Extra uterine conceptions lodged between the uterus and rectum. 6. Organic disease in the anterior wall of the rectum. 7. Stricture of the rectum. It is scarcely necessary to add, that the principal means relied upon for diagnosis is the introduction of the uterine sound.

"5. That the treatment should comprise the three following indications:—1. The removal, if necessary and possible, of any morbid action in the uterine structures that may exist along with the displacement. 2. The restoration of the uterus to its normal situation. 3. The use of means to retain it in its replaced and natural position. The first indication points to the removal of congestion, inflammation, and hypertrophy of the uterus—constriction of the cervix uteri; and painful, congestive, and inflammatory conditions of one or both ovaries. The second to the restoration of the uterus to its normal situation, which is best effected by introducing the uterine sound into the cavity of the organ, and using it as a lever. The retention of the replaced uterus in its normal situation is proposed to be accomplished by means of an intra-uterine pessary, which is to be worn for a period varying from one or two weeks to many months."

No part of Dr. Simpson's work will be more severely criticised than the paper setting forth these views. In the first place, it may be doubted whether the displacement referred to is productive of all the distressing sufferings and ailments ascribed to it by our author. "But," remarks one of his reviewers, "if the pathological nature and relations of a lesion are doubtful, it becomes still more necessary to be cautious in the application of our therapeutical or curative measures; and here we cannot help thinking that the means suggested for its cure are scarcely warranted by our knowledge of the nature of the disease. It is proposed by Dr. Simpson, first, to replace the organ by means of the uterine sound, and secondly, to fix and retain it in position by means of an intra-uterine pessary—that is to say, by means of an inflexible and unyielding instrument, which can permit of little or no uterine movement. Now, apart from the difficulty and danger which are said to have attended this proceeding, we are prepared to oppose the practice upon purely physiological grounds. We have already adverted to the fact that the uterus was never intended to hold a fixed and undeviating position, but to accommodate itself somewhat to the varying states of adjacent organs. In the words of Dr. Simpson, its position is capable of being changed to a very considerable extent without incommodity or injury, by such exterior influences as may naturally or accidentally act upon it. Its position is so far constantly changed by the varying states of distension of the bladder and rectum. Under voluntary efforts of straining it can in general be readily pushed down half an inch or an inch into the cavity of the vagina, and it may be drawn down by instruments till the cervix reaches the external parts themselves, or even protrudes beyond them. Now we contend that to fix an organ which nature never intended to be fixed, and to do away with all those necessary movements by which the uterus is enabled to accommodate itself to the varying conditions and necessities of neighboring organs, is not only to act in opposition to all sound physiological principles, but to adopt a practice which, apart from its dangers, must be productive of very doubtful advantages."

A number of shorter but highly interesting papers follow, some of which are on gout of the uterus, position of the patient for tapping in ovarian dropsy, injection of iodine into ovarian cysts, ovariotomy, its justification, amenorrhœa from imperfect development of the uterus, dilatation and incision of the cervix uteri in dysmenorrhœa, direct application of remedies to the cavity of the uterus, spurious pregnancy, and alleged infecundity of females born co-twins with males.

In regard to this latter question he closes his paper with the following remarks:

"The whole inquiry detailed in the few preceding pages forms an apt illustration of an old remark, that in medicine it often requires a much greater extent of observation and research to disprove satisfactorily an alleged and accredited fact, than was ever expended, either upon the original development or subsequent confirmation of it. In the present instance, the results have turned out to be perfectly contradictory of the opinion which I. in common with others, held regarding the infecundity of the female in double sexed twins, when I commenced looking into the subject; and instead of finding my preconceived ideas confirmed by the investigation, they have on the other hand, been completely confuted by it. For the data that I have adduced do, so far as they go, evidently prove—

"1. That, in the human subject, females born co-twin with males are, when married, as likely to have children as any other females belonging to the general community.

"2. That, when they are married and become mothers, they are, in respect to the number of their children, as productive as other females.

"3. That the same law of the fecundity of the female in opposite sexed twins seems to hold good among all our uniparous domestic animals, with the exception of the *cow* alone.

"Indeed, the strong confirmatory evidence which the preceding inquiry affords of this last exceptional point constitutes one of its most interesting results. For certainly it cannot but be considered as an extraordinary circumstance, that, in the cow, the twin existence in utero of a male along with a female should, as a general principle, lead—

"1. To so great a degree of malformation as we have described, in the sexual organs, and in the sexual organs only.

"2. That this malformation should be limited entirely to the reproductive organs of the female twin, while those of the male twin are perfectly and fully developed.

"3. That this sexual malformation should, apparently so far as we yet know, occur in the case of twins in the cow only, and in this species of uniparous animal alone. The curiosity of the fact becomes heightened and increased when we recollect that when the cow has both twins of the same sex, as two males or two females, these animals are always both perfectly formed in their sexual organization, and both capable of propagating. The whole series of circumstances, when considered in conjunction with each other, seems to form, in relation to the origin of malformations, one of the strangest and most inexplicable facts to be met with in the study of anormal development."

This brings us to the second part of the work, embracing the Physiology and pathology of Pregnancy; the first paper treating of the duration of pregnancy, its frequent irregularity, and occasional protraction. Dr. Simpson quotes a number of obstetricians to the point, that pregnancy has a fixed unvarying period, but what that period is the accouchers are not agreed among themselves. Sir Charles Clark assigns 40 weeks, or 280 days, as the duration, while Dr. Gooch places it at 273 to 275 days. Dr. Simpson believes that deviations from the normal period, both in the way of diminution and of excess of time are more common than is generally supposed among the members of the profession at large, and he reports two cases in which the pregnancy exceeded 280 days by several weeks, and shows by tables prepared by Merriman, Murphy, and Reid that it has been frequently protracted to the 295th, 302d, and even the 326th day. Similar tables show that the period varies as much in cows as in the human female. Thus Lord Spencer reports the date of delivery in seven hundred and fifty-four cows in which the date of impregnation had been carefully registered, and of these the period extended to the 302d and 321st day. M. Tessier had previously made five hundred and seventy-two observations of a similar kind upon the same animal, and with corresponding results. The result of the whole is that the period varies, both in the cow and the human female, from the 37th to the 44th week, and upwards. The protraction in some cases is habitual, the woman going regularly beyond her time, and in other instances it seems to be hereditary, the daughter inheriting the peculiarity of the mother.

Why parturition should take place with certainty at a given period, while dentition, puberty, menstruation, etc., are known to vary so greatly, no one has attempted to explain. The same irregularity ought to be expected in this process that is acknowledged te prevail in the others. Dr. Simpson assigns a cause for parturition which strikes us as more satisfactory than any hitherto proposed. He ascribes it to "changes going on, or rather accomplished, between the uterus and its deciduous lining—which changes lead to parturition, when they have proceeded so far as to effect the necessary amount of disintegration and separation between the relatively attached surfaces of the uterus and decidua." These changes he deems to be "of a nature analogous to the so-called fatty degeneration, which occurs in effete and worn-out structures in other parts of the body." The time of this degeneration will vary in different individuals and under different circumstances, and hence the irregularity of the period of utero-gestation, as exhibited by the tables of Merriman, Murphy, Reid, Spencer, and Tessier.

In several succeeding papers, in Part III. of his Contributions, Dr. Simpson treats of the mechanism of natural labor, uterine inertia as a cause of tedious labor, and galvanism as a means of increasing the parturient contractions of the uterus. As the result of numerous trials with this agent, he concludes that galvanism has no efficacy as a stimulant to the expulsive action of the uterus.

In his next paper Dr. Simpson shows that the sex of the child exerts a manifest influence upon the mortality of mother and child. His conclusions, drawn from extended researches, may be stated in the following propositions:—

1. Of the mothers that die under parturition and its immediate consequences, a much greater proportion have given birth to male than female children.

2. Among labors presenting morbid complications and difficulties, the child is much oftener male than female.

3. Among the children of the mothers that die from labor or its consequences, a larger proportion of those that are still-born are male than female; and on the contrary, of those that are born alive, a larger proportion are female than male.

4. Of still-born children a larger proportion are male than female.

5. Of the children that die during the actual progress of parturition, the number of males is much greater than the number of females.

6. Of those children that are born alive, more males than females are seen to suffer from the morbid states and injuries resulting from parturition.

7. More male than female children die in the earliest periods infancy; and the disproportion between the mortality of the two sexes gradually diminishes from birth onwards till sometime subsequently to it.

8. Of the children that die in utero, and before the commencement of labor, as large a proportion are female as male.

9. In "laborious labors," with the head presenting in proportion as the order of labor rises in difficulty, the amount of male births in them rises in number.

10. Of the morbid accidents that are liable to happen in connection with the third stage of labor, as many take place with female as with male births.

11. More dangers and deaths occur both to mothers and children in first than in subsequent labors.

12. The average duration of labor is longer with male than with female children.

He concludes from all the calculations he has made, that "there have been lost in Great Britain during a period of a little more than seven years, as a consequence of the slightly larger size of the male than of the female head at birth, about 50,000 lives including those of about 46,000 or 47,000 infants, and of between 3,000 and 4,000 mothers who have died in child-birth."

We pass over two elaborate papers, one on the irregularities of head presentations, the other on the treatment of face presentations, as also over some shorter articles, to notice his memoir on turning as a substitute for craniotomy. Dr. Simpson sums up thus the advantages which, in regard to the mechanism of labor, are, in the contracted states of the pelvic brim, obtained by the child passing as a footling instead of as a cephalic presentation:—

"1. The fœtal cranium is of a conical form, enlarging from below upwards, and when the child passes as a footling presentation, the lower and narrower part of the cone-shaped head is generally quite small enough to enter and engage in the contracted pelvic brim.

"2. The hold which we have of the protruded body of the child, after its extremities and trunk are born, gives us the power of employing so much extractive force and traction at the engaged fœtal head, as to make the elastic sides of the upper and broader portion of the cone (viz: the bi-parietal diameter of the cranium) become compressed, and, if necessary, indented, between the opposite parts of the contracted pelvic brim, to such a degree as to allow the transit of the entire volume of the head.

"3. The head in being dragged downwards into the distorted pelvis, generally arranges itself, or may be artificially adjusted, so that its narrow bi-temporal, instead of its bi-parietal, diameter, becomes engaged in the most contracted diameter of the pelvic brim.

"4. The arch of the cranium or head is more readily compressed to the flattened form and size required for its passage through a contracted brim, by having the compressing power applied, as in footling presentations and extraction, directly to its sides or lateral surfaces, than by having it applied, as in cephalic presentations, partly to the lateral and partly to the upper surfaces of the arch.

"Lastly, I may add, as a result of the whole mechanism, that the *duration* of the efforts and sufferings of the mother is greatly abridged by turning, when used as an alternative for craniotomy and the long forceps, and that thereby her chances of recovery and safety are increased But as this is in itself a matter of the highest moment, in reference to the whole question of the proposed practice, we shall devote a special section to the consideration of it."

In cases where the pelvis is somewhat too small, or the fœtal head somewhat too large, to allow the infant to pass by the unaided efforts of nature, or even with the assistance of the long forceps, if that instrument is had recourse to—the cases in which Dr. S. recommends turning instead of craniotomy—the following are the advantages gained by the procedure:—

"1. It substitutes the delivery of the infant by the hand of the accoucheur, for its delivery by formidable steel instruments. And, certainly, the avoidance of instruments is, as a general principle, desirable when it is possible.

"2. The transit of the cone-shaped head of the child through a somewhat narrow brim is facilitated by the narrow end of the cone (or bi-mastoid diameter of the head) being made to enter and engage first in the contracted brim; and the hold which we obtain of the extruded body of the child enables us to employ so much extractive force upon the engaged fœtal head, as to make the elastic sides of the upper and broader portion of the cone (or bi parietal diameter of the cranium) to become compressed, and if necessary indented, between the sides of the contracted brim.

"3. When the child is brought down footling, we have far more power than when the spherical arch of the cranium presents, of manually adapting and adjusting, when necessary, the shape of the head to the shape of the contracted brim; the rounded form of the cranium not affording us any sufficient hold and purchase for this purpose in cranial presentations.

"4. The *lateral* and very *temporary* compression of the fœtal head by the contracted sides of the pelvis, such as we can produce and effect on artificial turning and contraction, is less dangerous to the life of the child than its *oblique* or longitudinal compression with the long forceps, or by the *long* impaction of the head itself in the contracted brim.

"5. In cases where the narrowness is greater, and such as to produce a depression or indentation in the elastic and flexible cranium of the child, still this transient depression, or indentation, is not necessarily destructive to life, as the perforation of the head in craniotomy. Children often revive and recover when born with the head much distorted and even indented. See, for example, the child in Case II., and other similar instances recorded by Smellie, Denman, Velpeau, Duges, Jacquemier, Radford, etc., etc.

"6. On these accounts the operation of turning affords a fair chance of life to the child, while craniotomy affords none. And even when the turning and extraction require some considerable time for their performance, the resulting temporary asphyxia of the child is not necessarily so deep and fatal but that the infant may be revived by appropriate measures applied after birth. I can, for one, state that in these cases, and in instances of common footling and turning cases, I have been repeatedly astonished at the viability of the infant after traction had been applied to it, both so strong in degree and so long in duration as to leave apparently little hope of its survival; and I have heard other practitioners make the same remark as the result of their experience.

"7. The operation of turning, under the circumstances we speak of, will, I believe, be found also to be more safe to the life of the mother than the operation of craniotomy. In every instance the operation of craniotomy is necessarily fatal to the infant; but in a very large proportion also, this operation is fatal to the mother. The statistical results collected by Dr. Churchill and others show that craniotomy is fatal to the mother in about 1 in every 5 cases in which it is performed; while turning does not generally prove fatal in above 1 in every 15 or 16 patients, even including complicated cases. Besides, it affords this great source of safety to the mother, that, *cœteris paribus*, delivery by turning can be, and is, as a general rule, adopted far earlier in the labor than delivery by craniotomy; and in proportion as it is practised earlier, so far also will it be practised with greater safety and greater success—the maternal mortality attendant upon parturition, whether natural or operative, increasing always in a ratio progressive with the increased duration of the labor."

The only other memoir that we have room to notice is one of the ablest in the volume—the paper on the extraction of the placenta before the child in cases of placental presentation, which he begins with a consideration of the dangers of such presentations as evinced by statistics, and with a statement of the opinions of authors on the subject. The ground on which Dr. Simpson rests the propriety of extracting the placenta before the child in these alarming cases may be briefly stated to be, that when the placenta is separated completely the hemorrhage is less than where there is only a partial separation, and is, in fact, exceedingly slight and trifling. In proof of this position he relates numerous cases in which the placenta was expelled before the child without the occurrence of hemorrhage, and constructs a table of 141 cases in which the expulsion of the placenta preceded the birth of the child with but a slight loss of blood in a great majority of instances. He sums up the results of his analysis of these cases in the following propositions:—

"1. The complete separation and expulsion of the placenta before the child, in cases of unavoidable hemorrhage, is not so rare an occurrence as accoucheurs appear generally to believe.

"2. It is not by any means so serious and dangerous a complication as might *à priori* be supposed.

"3. In nineteen out of twenty cases in which it has happened, the attendant hemorrhage has either been at once altogether arrested, or it has become so much diminished as not to be afterwards alarming.

"4. The presence or absence of flooding after the complete separation of the placenta, does not seem in any degree to be regulated by the duration of time intervening between the detachment of the placenta and the birth of the child.

"5. In ten out of one hundred and forty-one cases, or in one out of fourteen, the mother died after the complete expulsion or extraction of the placenta before the child.

"6. In seven or eight out of these ten casualities, the death of the mother seems to have had no connection with the complete detachment of the placenta, or with results arising directly from it, and if we do admit the three remaining cases, which are doubtful, as leading by this occurrence to a fatal termination, they would still only constitute a mortality from this complication, of three in one hundred and forty-one, or of about one in forty-seven cases.

"7. On the other hand, under the present established rules of practice, one hundred and eighty mothers died in six hundred and fifty-four placental presentations, or nearly one in three."

We quote Dr. S.'s remarks on what he regards as the cause of the cessation of the hemorrhage under such circumstances:—

"If the explanation which I have above given of the source of the hemorrhage in partial detachment of the placenta, be true—namely, that it proceeds principally, if not entirely, from the maternal vascular cells belonging to the separated portion of the organ being still, more or less freely, supplied through the utero-placental vessels of the adhering portion—we can further easily understand how it occurs that the attendant hemorrhage is immediately moderated, or entirely arrested, when the placenta is once thoroughly and perfectly separated from the interior of the uterus, as in the class of cases which form the subject of the present memoir. If the flooding proceeds, as I have endeavored to show, from the detached and exposed surface of the placenta, and not from the detached and exposed surface of the uterus, the placenta must cease to yield any new or additional quantity of maternal blood, as soon as its own vascular connections with the mother are destroyed; or, in other words, the immediate source of supply of the hemorrhage is cut off, and its continuance consequently prevented, as soon as the placenta is entirely separated from the interior of the uterus.

"Besides the preceding *anatomical* considerations, we must take some important *physiological* points into consideration, in investigating the mechanism of the complete cessation of unavoidable hemorrhage upon the complete separation of the placenta. The uterus, during pregnancy, has, like other organs under high vital periodic activity, both its arteries and veins, but especially the latter, enormously enlarged. The immediate and final object of this great enlargement is to supply the necessary materials for nourishment and respiration to the included fœtus. The medium through which these materials are supplied to the fœtus is the placenta; and the maternal cells of that organ form the more immediate locality in which they are transmitted from the maternal to the fœtal system. We have already stated that these maternal placental cells are merely dilated capillaries, inasmuch as they constitute the only vascular connection between the terminations of the utero-placental arteries and the origins of the utero-placental veins. The capillaries, especially of those parts and organs which are liable to periodic excesses of action, an increased determination of blood to themselves and their corresponding arteries and veins, and that quite independently of any change whatever in the central organ of the circulation.* This seems to hold true to a great and remarkable degree with regard to the maternal form of capillary circulation carried on in the placenta, in consequence of the great and remarkable functions which this temporary portion of capillary circulation is destined to perform. And if the organic relations of the placenta to the uterus are disturbed, the resulting deviations are equally striking. If the placenta is only partially detached, the 'attractive' power of the maternal circulation in the organ is, with the other moving powers of the blood, generally sufficient to keep the cells of the detached, as well as those of the adherent portion, filled with blood; and at the same time the circulation in the uterine arteries and veins in the immediate neighborhood continues to be more or less vigorously maintained, in consequence of their contiguity to the free tide of communication that is carried on between the uterine vessels, and the part of the placenta that still remains affixed. Hence, probably, even the orifices of the congested and enlarged uterine veins opening on the interior of the uterus, and lying near the existing line of junction between the uterus and the placenta, may occasionally allow of some discharge, in addition to the freer form of flooding that we have seen to take place from the more patulous apertures left on the exposed surface of the placenta itself. But separate entirely and completely the placenta from the uterus, and then you at once alter the course—as you have at once removed the great physiological aim and object—of utero-placental circulation. The blood in the uterine vessels being now no longer attracted by the maternal capillary system of the placenta, and so far the *distributive* influence of that system being completely and suddenly abrogated, there is not only a less absolute tide of blood determined towards the uterus, but that which is contained in its vessels seeks the freest and most potent course backward to the heart through the higher and larger communicating branches between the uterine arteries and veins. The placental capillary influence no longer *turns aside* from these channels, the direct and onward course of the circulating current into its own set of special uterine vessels. These special vessels, now that their function is arrested, are comparatively empty of blood, and their collapsing or collapsed sides may serve to keep the general vascular current in its proper canals; for the hemorrhage, if any, is from venous orifices, and hence easily repressed by slight impediments; and we shall afterwards see that it is not a direct discharge, but arises from retrogression or regurgitation of the blood in the venous tubes."

* "The capillaries possess a distributive power over the blood, so as at least to regulate the local circulation, independently of the central organ, in obedience to the necessities of each part."—See note in Mr. Palmer's admirable edition of the Works of John Hunter, vol. iii., p. 332.

Dr. Simpson would not be understood as recommending this practice in all cases of placenta praevia. On the contrary, there are cases in which the patient may be safely left to nature after a partial separation of the placenta; as where the hemorrhage is slight, or ceases spontaneously; but such cases, Dr. S. thinks, are rare. The only other modes of affording relief in such circumstances are the evacuation of the liquor amnii, or the artificial extraction of the child. When they are inapplicable or unavailing the artificial separation of the placenta must be resorted to when the combination of circumstances is as follows:—

"The hemorrhage is so great as to show the necessity of interference, and is not restrained or restrainable by milder measures, such as the evacuation of the liquor amnii; but, at the same time, turning, or any other mode of immediate and forcible delivery of the child, is especially hazardous or impractiable, in consequence of the undilated or undeveloped state of the os uteri, the contraction of the pelvic passages, etc. Or, again, the death, prematurity, or non-viability of the infant, may not require us to adopt modes of delivery for its sake, that are accompanied (as turning is) with much peril to the mother, provided we have a simpler and safer means, such as the detachment of the placenta, for at once commanding and restraining the hemorrhage, and guarding the life of the parent against the dangers of its continuance. Hence, as I have elsewhere stated, I believe that the suppression of the flooding by the total detachment of the placenta will be found the proper line of practice in severe cases of unavoidable hemorrhage, complicated with an os uteri so insufficiently dilated and undilatable as not to allow of version being performed with perfect safety to the mother: therefore, in most primiparæ; in many cases in which placental presentations are, as very often happens, connected with premature labor and imperiect development of the cervix and os uteri; in labors supevening earlier than the seventh month; when the uterus is too contracted to allow of turning; when the pelvis or passages of the mother are organically contracted; when the child is dead; when it is premature and not viable; and where the mother is in such an extreme state of exhaustion as to be unable, without immediate peril of life, to be submitted to the shock and dangers of turning, or forcible delivery of the infant. This enumeration is far from comprehending all the forms of placental presentations that are met with in practice; but it certainly includes a considerable proportion of the cases of this obstetric complication, and among them all, or almost all, of the most dangerous and most difficult varieties of unavoidable hemorrhage. In adopting the practice, one error, which I would strongly protest against, has been committed in some instances. Besides completely detaching and extracting the placenta, the child has subsequently been extracted by direct operative interference. If the hemorrhage ceases, as it usually does, upon the placenta being completely separated, the expulsion of the child should be subsequently left to nature, unless it present preternaturally, or the labor afterwards show any kind of complication which of itself would require operative interference under any other circumstances. Both to detach the placenta and extract the child would be hazarding a double instead of a single operation."

In taking leave of this work, so tasteful and pleasing in its appearance, we need hardly, after the numerous extracts we have given, reiterate the very favorable opinion which we expressed at the outset of its scientific and literary merits. It abounds in matter of the deepest interest to the practitioner, and is written in a style at once forcible, scholarly, and clear. How high soever the estimate in which the reader may have held the professional learning, ingenuity, and ability of the author, we venture to say that that estimate will be raised by the perusal of these Memoirs. It is a work which is sure to find its way into the libraries of most of our readers, and to become one of the standard works of the profession. The publishers have executed their part of the enterprize in a manner worthy of the high scientific character of the volume.

